# Metagenomics and Quantitative Stable Isotope Probing Offer Insights into Metabolism of Polycyclic Aromatic Hydrocarbon Degraders in Chronically Polluted Seawater

**DOI:** 10.1128/mSystems.00245-21

**Published:** 2021-05-11

**Authors:** Ella T. Sieradzki, Michael Morando, Jed A. Fuhrman

**Affiliations:** a Department of Biological Sciences, University of Southern California, Los Angeles, California, USA; University of Hawaii at Manoa

**Keywords:** metagenomics, aromatic hydrocarbons, bioremediation, marine microbiology, microbial ecology, stable isotope probing

## Abstract

Bacterial biodegradation is a significant contributor to remineralization of polycyclic aromatic hydrocarbons (PAHs)—toxic and recalcitrant components of crude oil as well as by-products of partial combustion chronically introduced into seawater via atmospheric deposition. The Deepwater Horizon oil spill demonstrated the speed at which a seed PAH-degrading community maintained by chronic inputs responds to acute pollution. We investigated the diversity and functional potential of a similar seed community in the chronically polluted Port of Los Angeles (POLA), using stable isotope probing with naphthalene, deep-sequenced metagenomes, and carbon incorporation rate measurements at the port and in two sites in the San Pedro Channel. We demonstrate the ability of the community of degraders at the POLA to incorporate carbon from naphthalene, leading to a quick shift in microbial community composition to be dominated by the normally rare *Colwellia* and *Cycloclasticus*. We show that metagenome-assembled genomes (MAGs) belonged to these naphthalene degraders by matching their 16S-rRNA gene with experimental stable isotope probing data. Surprisingly, we did not find a full PAH degradation pathway in those genomes, even when combining genes from the entire microbial community, leading us to hypothesize that promiscuous dehydrogenases replace canonical naphthalene degradation enzymes in this site. We compared metabolic pathways identified in 29 genomes whose abundance increased in the presence of naphthalene to generate genomic-based recommendations for future optimization of PAH bioremediation at the POLA, e.g., ammonium as opposed to urea, heme or hemoproteins as an iron source, and polar amino acids.

**IMPORTANCE** Oil spills in the marine environment have a devastating effect on marine life and biogeochemical cycles through bioaccumulation of toxic hydrocarbons and oxygen depletion by hydrocarbon-degrading bacteria. Oil-degrading bacteria occur naturally in the ocean, especially where they are supported by chronic inputs of oil or other organic carbon sources, and have a significant role in degradation of oil spills. Polycyclic aromatic hydrocarbons are the most persistent and toxic component of crude oil. Therefore, the bacteria that can break those molecules down are of particular importance. We identified such bacteria at the Port of Los Angeles (POLA), one of the busiest ports worldwide, and characterized their metabolic capabilities. We propose chemical targets based on those analyses to stimulate the activity of these bacteria in case of an oil spill in the Port POLA.

## Abstract (precis)

Oil spills in the marine environment have a devastating effect on marine life and biogeochemical cycles through bioaccumulation of toxic hydrocarbons and oxygen depletion by hydrocarbon-degrading bacteria. Oil-degrading bacteria occur naturally in the ocean, especially where they are supported by chronic inputs of oil or other organic carbon sources, and have a significant role in degradation of oil spills.

## INTRODUCTION

Polycyclic aromatic hydrocarbons (PAHs) are recalcitrant, mutagenic, and carcinogenic components of fossil fuel as well as by-products of incomplete combustion but are also part of cosmic dust, hydrothermal vent plumes, and algae (1, 2). Microbial biodegradation has an important role in PAH remediation alongside physical weathering processes (3). Biodegradation of PAHs captured much scientific attention after the Deepwater Horizon (DWH) oil spill in the Gulf of Mexico in 2010. Several studies measured PAH degradation rates (4, 5) and showed enrichment of known PAH-degrading bacteria in beaches, surface water, the deep-sea plume, and sediments even months after the spill began (6–11). Bacteria known to have the ability to utilize PAHs as a carbon source include strains of *Cycloclasticus*, *Colwellia*, Pseudomonas, *Alteromonas*, and others (7, 12–14). Many coastal sites worldwide experience chronic input of PAHs, mainly from atmospheric deposition and natural oil seeps. Recent studies show that chronic pollution supports a consistent “seed” of PAH-degrading bacteria which can respond quickly to acute pollution such as an oil spill (7, 12–18). However, seed communities of PAH-degrading bacteria are ubiquitous in the world’s oceans and are thought to be supported by biogenic, geological, and extraterrestrial PAH sources (2).

Stable isotope probing (SIP) is a well-established method for the identification of environmental bacteria utilizing targeted substrates, in this case PAHs (19, 20). ^13^C-labeled PAHs are added to samples and incorporated into the DNA of PAH-utilizers, causing its density to increase. Heavier DNA can then be physically separated in a density gradient. A large-scale SIP study was performed on DWH surface and deep-plume water, revealing local strains of PAH-degrading bacteria that responded to the input of hydrocarbons (7). Some genomes of those bacteria were assembled from mesocosm metagenomes in order to further explore their PAH metabolism (21). The studies mentioned above, like many others, focus only on the high-density (i.e., most heavily ^13^C-labeled) fractions under the assumption that the most heavily labeled organisms, and thus the main targets, will be found there. However, this strategy may lead to overlooking degraders with low-GC (i.e., naturally lower DNA density) genomes, whose DNA may not appear in the heaviest fractions even if they include moderate amounts of ^13^C (22, 23). Tag-SIP is a powerful and particularly sensitive extension of the standard SIP approach, in which DNA from both labeled samples and parallel unlabeled controls are separated into density fractions, and the 16S-rRNA gene is amplified from all density fractions of both samples for comparison. This approach, circumventing GC-based bias, allows us to track substrate incorporation by each single taxon demonstrated by an increase (shift) in its DNA density in the labeled samples compared to controls (24, 25).

One of the main motivations to study PAH-degrading organisms is to characterize their metabolic requirements. An understanding of the suite of nutrients and cofactors those organisms require could potentially be applied toward bioremediation and biostimulation (26). The combination of SIP with metagenomics can help reveal metabolic dependencies within assembled genomes of PAH-degraders (3, 17, 21, 26). Several microorganisms have been demonstrated to degrade PAHs in axenic culture (27–29), and degradation pathways have been identified based on their genomes. Culture-defined naphthalene degradation begins with hydroxylation of one of the rings by naphthalene-1,2-dioxygenase, which is considered the rate-limiting step. Further oxidation steps lead to generation of catechol, gentisate, homogentisate, or protocatechuate, by-products into which many aromatic degradation pathways are funneled (30). These by-products are degraded, and their products are incorporated into cellular metabolism. While single organisms can degrade PAHs, it has also been proposed that PAH biodegradation in the environment could be a community process (21), likely influenced by interactions such as competition, predation, and effects of abiotic factors absent from culture studies. Thus, it is important to identify not only the degradation enzymes and nutrient requirements present in primary degraders, but also those in other members of the community which could have a more minor role in PAH degradation.

Here, we hypothesized that rare microbial taxa (<0.001% of the community) would be major incorporators of ^13^C-from PAHs. Naphthalene was used as a model substrate to select for communities involved in catabolism of PAHs (31). We then assembled and mined information from genomes of primary and potential secondary degraders on their metabolic requirements and proposed targets for biostimulation experiments in this system.

## RESULTS

### Naphthalene uptake rates across the San Pedro Channel.

**FIG 1:**
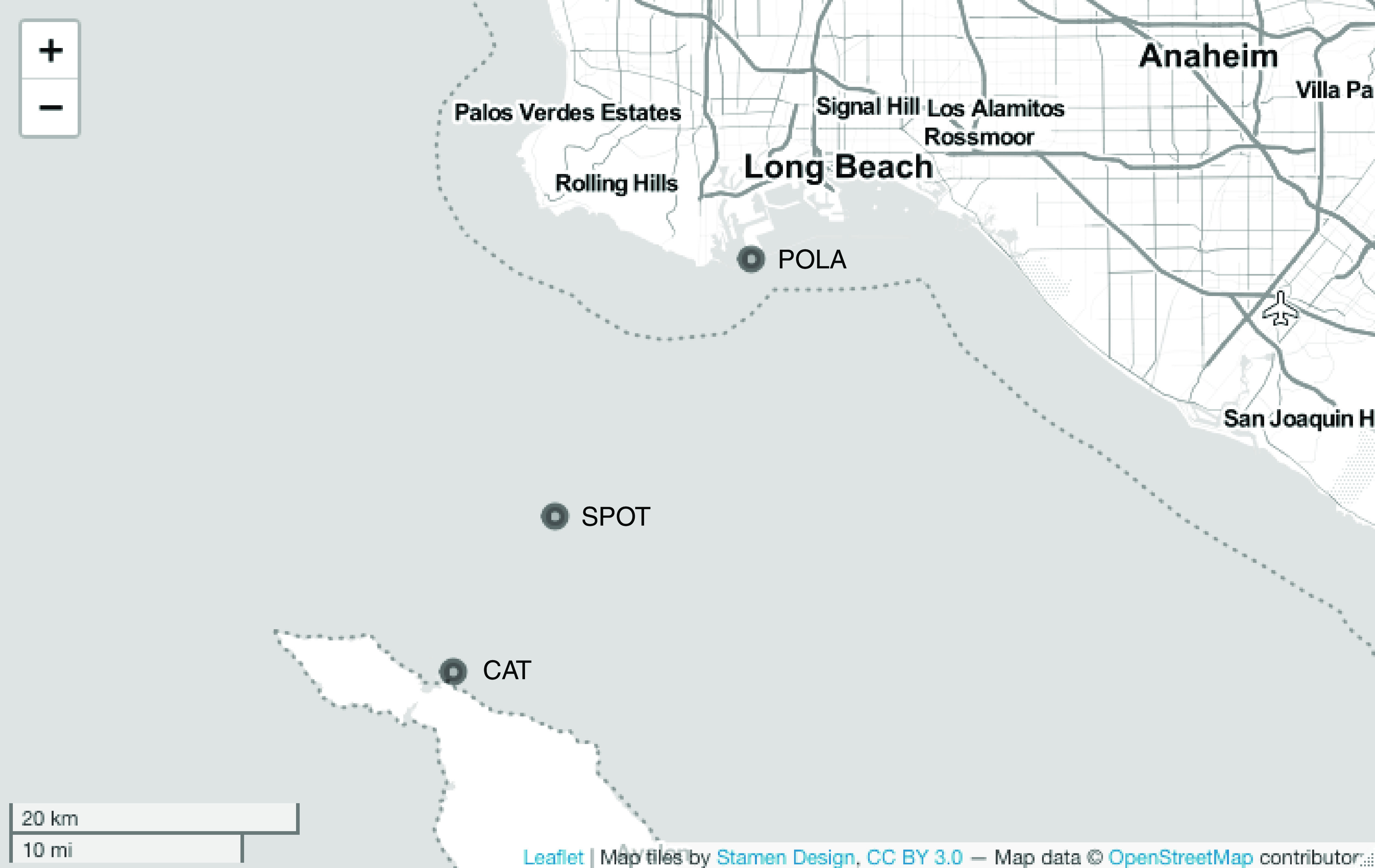
Map of the sampling sites across the San Pedro Channel; the Port of Los Angeles (POLA), the San Pedro Ocean Time-series (SPOT), and Santa Catalina Island (CAT). The sites are within a range of 40 km. This map was created using leafletR (99).

Our main hypothesis was that at the Port of Los Angeles (POLA) there would be a community of PAH-degrading bacteria likely sustained by chronic inputs and eutrophication. One indication of the existence of such a community would be measurable uptake of naphthalene-derived carbon upon amendment with PAHs, in this case naphthalene. We also wanted to test whether this degradation potential extended out into the San Pedro Channel at the San Pedro Ocean Time-series (SPOT) and Two Harbors (CAT) (Fig. 1). Isotopic enrichment measurements at POLA indicated a mean naphthalene uptake rate of 35 nM/day (standard deviation, 19.53 nM/day) given a high input of 400 nM naphthalene. However, naphthalene incorporation rates at SPOT and CAT were below detection.

### PAH-degrading taxa at POLA identified by SIP.

**FIG 2:**
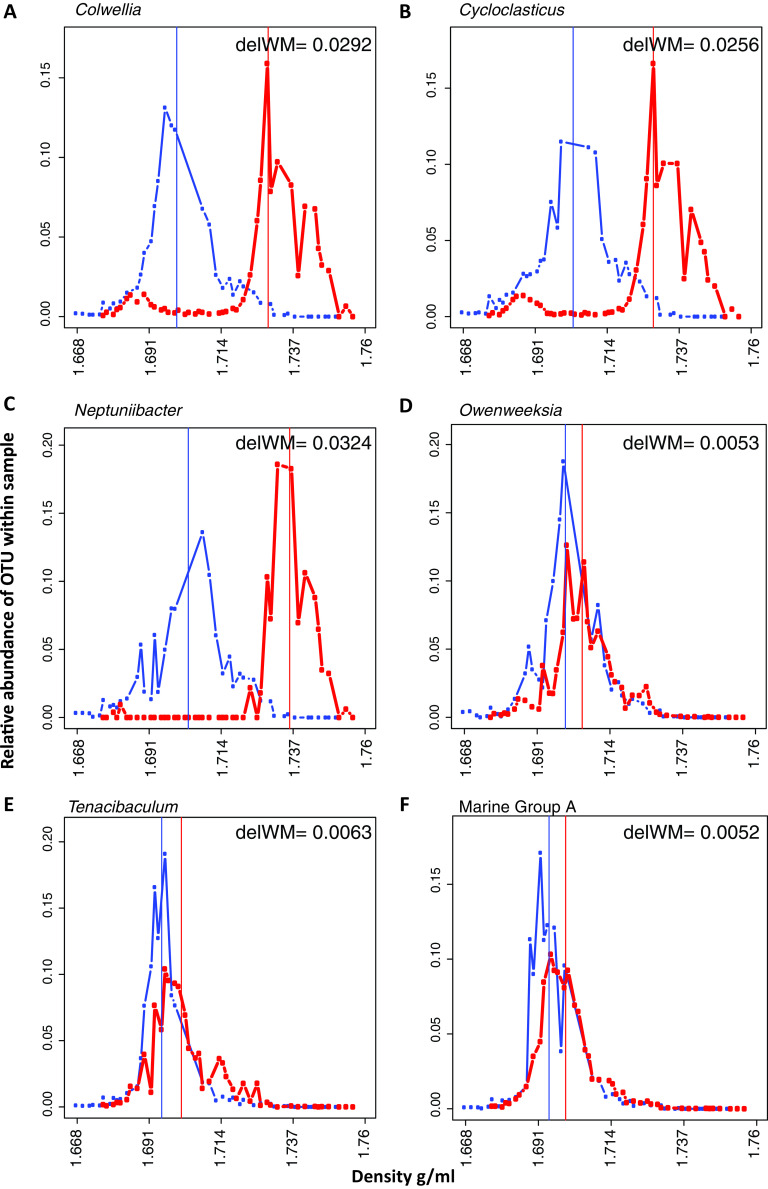
Density shifts demonstrating which taxa took up ^13^C naphthalene. (A to F) Distribution of labeled (^13^C, red) and control (^12^C, blue) normalized relative abundance as a function of buoyant density of (A) *Colwellia*, (B) *Cycloclasticus*, (C) *Neptuniibacter*, (D) *Owenweeksia*, (E) *Tenacibaculum*, and (F) Marine group A. Vertical lines represent the weighted mean of the distribution. The difference between the weighted mean density (WM) of the labeled (^13^C) and control (^12^C) (delWM) is noted on each plot. OTUs are based on 16S-rRNA community analysis performed on each fraction normalized by the amount of DNA per fraction.

The ^13^C-naphthalene-enrichment of POLA seawater led to significant incorporation of labeled carbon (>0.005 g · ml^−1^ buoyant density increase corresponding to 9 atom percent excess) by 34 out of 180 operational taxonomic units (OTUs) (Data set S2). After 88 h of incubation *Colwellia* spp. and *Cycloclasticus* spp. (*Gammaproteobacteria*) made up 40% of the planktonic (0.2 to 1 μ) microbial community. These OTUs were enriched at 53 (*Colwellia*) and 47 (*Cycloclasticus*) atom percent excess (32) (Fig. 2A and B). These main naphthalene degraders were rare (<0.2% cumulative relative abundance of all OTUs classified as *Colwellia* or *Cycloclasticus*) to nondetectable prior to enrichment (t_0_) at all sites on all dates. While both taxa were represented by multiple OTUs, there was always a dominant OTU which matched the 16S-rRNA genes from the metagenome-assembled genomes (MAGs; see below). The most abundant OTU accounted for 95% and 99% of the *Colwellia* and *Cycloclasticus* amplicons, respectively. Additional significantly enriched OTUs included *Gammaproteobacteria* (*Marinomonas*, *Neptuniibacter*, *Porticoccus*, *Pseudoalteromonas*, SAR86, and *Vibrio*), *Flavobacteriales* (*Tenacibaculum*, *Fluviicola*, *Polaribacter*, NS7 Marine group, and *Owenweeksia*), *Sphingobacteriales*, *Deferribacterales* (Marine group A), and *Rhodospirillales* (Fig. 2C to F).

10.1128/mSystems.00245-21.7 DATA SET S2 General information on metagenomic assembled genomes (percent completion, percent redundancy, length), taxonomy determined using GTDB-Tk, Anvi’o, GToTree, and 16S-rRNA and mean coverage in metagenomes (second and third quartiles—Anvi’o setting Q2Q3). Download Data Set S2, XLSX file, 0.03 MB. Copyright © 2021 Sieradzki et al. 2021 Sieradzki et al. https://creativecommons.org/licenses/by/4.0/ This content is distributed under the terms of the Creative Commons Attribution 4.0 International license.

### Metagenome-assembled genomes (MAGs) from naphthalene-amended water.

**FIG 3:**
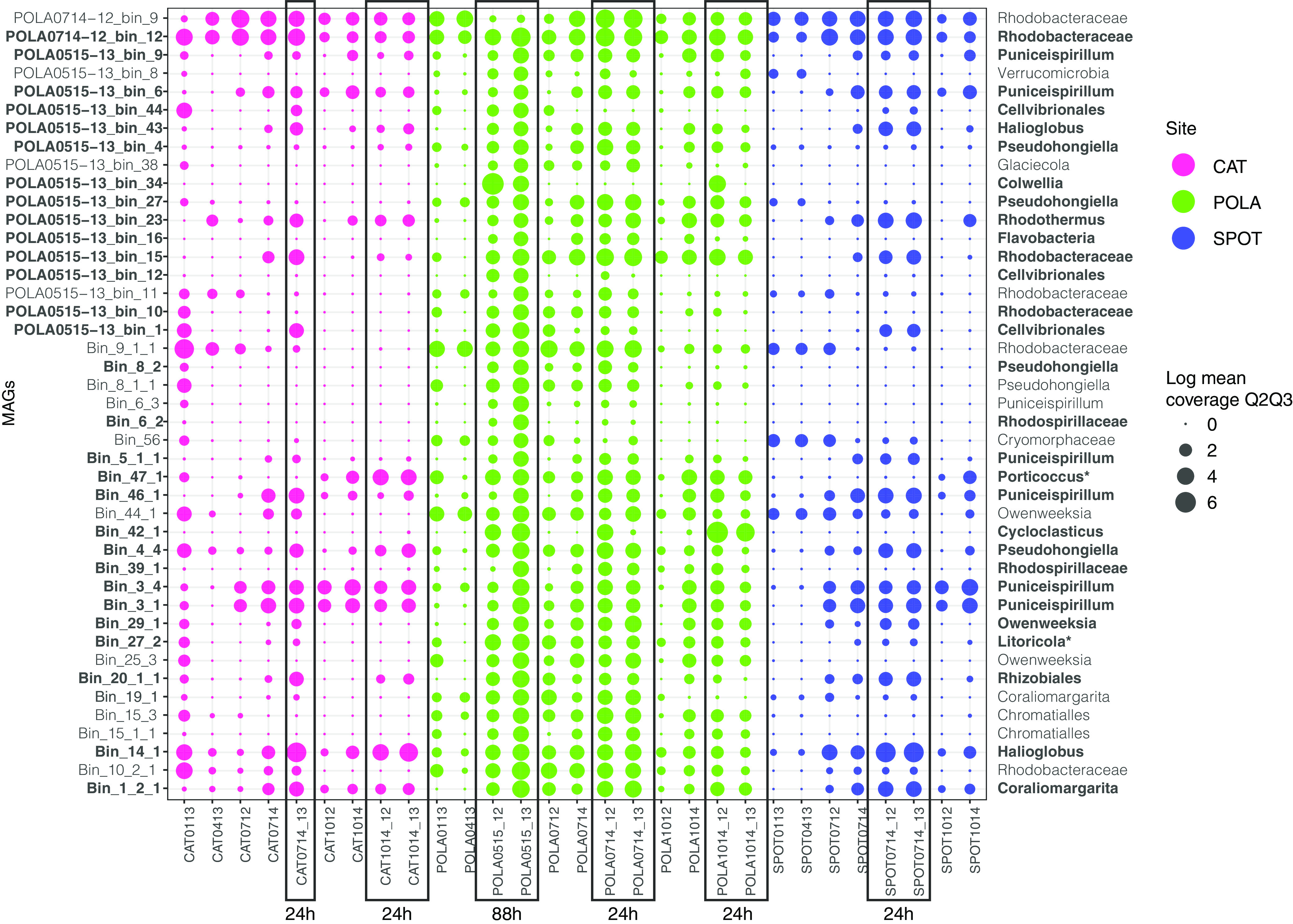
Abundance of MAGs in response to amendment with naphthalene. Each row represents a MAG. Mean metagenomic coverage (measure of abundance) of MAGs in naphthalene-amended and unamended seawater was normalized to sequencing depth and log-transformed. Each column represents a sample with collection month and year indicated (e.g., 0113 is January 2013). Amended samples have _12 (^12^C-naphthalene added) or _13 (^13^C-naphthalene added) next to the sample name and are outlined by rectangles. Incubation time is denoted under the rectangles. The rest of the samples represent t_0_ (before naphthalene addition). MAG taxonomy by GToTree is displayed on the right. MAGs in bold font had significantly higher coverage in naphthalene-amended samples (*P* value < 0.05). *, 16S-rRNA-based taxonomy where its resolution was higher than GToTree taxonomy.

We assembled and binned 43 dereplicated MAGs that were more than 50% complete (mean, 88%; standard deviation [SD], 11%) and less than 10% redundant (mean, 4.6%; SD, 2.6%) from naphthalene-amended POLA water. We then mapped reads from naphthalene-amended and unamended (t_0_) metagenomes to those MAGs in order to pinpoint potential degraders, under the assumption that potential degraders would be more abundant in amended metagenomes than in t_0_ metagenomes (Fig. 3). Interestingly, seven bins were enriched in the presence of naphthalene at SPOT and CAT—POLA0515-13_bin_43, POLA0515-13_bin_23, Bin_4_4, Bin_47_1, Bin_46_1, Bin_20_1_1, and Bin_1_2_1 (Fig. S3). Eight bins significantly increased in abundance only in naphthalene-amended POLA water (mean coverage, <1 in all CAT and SPOT samples) (Table 1).

**TABLE 1:** Taxonomy and genomic parameters of MAGs whose abundance increased at POLA upon naphthalene addition

MAG ID	Taxonomy	Completeness (%)	Redundancy (%)	Size (Mbp)
POLA0515-13_bin_34	*Colwellia*	91	3.6	3.5
Bin_42_1	*Cycloclasticus*	99	2.9	2.4
POLA0515-13_bin_16	Flavobacteria	99	3.6	2.7
POLA0515-13_bin_12	*Cellvibrionales*	89	0.7	2.3
POLA0515-13_bin_27	*Pseudohongiela*	93	10	2.8
POLA0515-13_bin_4	*Pseudohongiela*	61	4.3	3.8
Bin_39_1	*Rhodospirillaceae*	91	5	3.2
Bin_6_2	*Rhodospirillaceae*	96	4.3	3.8

10.1128/mSystems.00245-21.3 FIG S3 Mean coverage of MAGs with increased abundance in naphthalene-amended water from SPOT/CAT. Download FIG S3, PDF file, 0.1 MB. Copyright © 2021 Sieradzki et al. 2021 Sieradzki et al. https://creativecommons.org/licenses/by/4.0/ This content is distributed under the terms of the Creative Commons Attribution 4.0 International license.

### Primary naphthalene degraders.

Two MAGs of interest were classified as *Colwellia* sp. and *Cycloclasticus* sp. and contained 16S-rRNA genes that matched at 100% identity to the most abundant OTUs of PAH-carbon incorporators (Fig. 2A and B).

**FIG 4:**
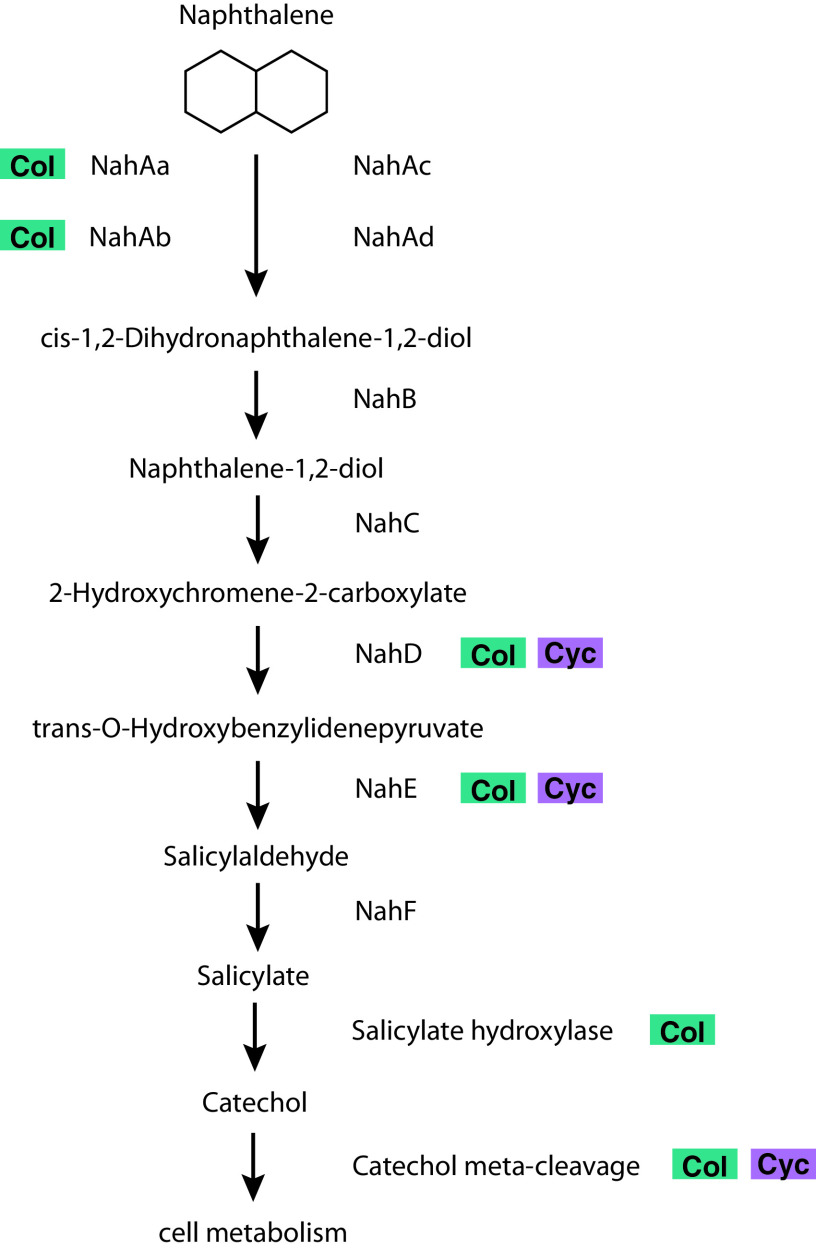
Simplified naphthalene degradation pathway and presence of genes coding for its enzymes in the *Colwellia* (Col, green) and *Cycloclasticus* (Cyc, purple) MAGs.

The *Colwellia* MAG had high coverage and breadth (portion of the MAG that has at least 1× coverage) only in naphthalene-amended POLA water from May 2015 and October 2014 (Fig. 3). Its closest relative based on a phylogenomic tree of 117 single-copy genes (33) is *Colwellia* sp. strain PAMC 20917 isolated from the Mid-Atlantic Ridge cold, oxic subseafloor aquifer (34) (Fig. S4; Data set S3). It has a 78% average nucleotide identity (two-way ANI) and 63% average amino acid identity (two-way AAI) with the *Colwellia* MAG assembled from the Deepwater Horizon oil spill (21). This MAG contained subunits A and B of the naphthalene dioxygenase enzyme (*nahAa*, *nahAb*), which is the first step in naphthalene degradation, but only parts of the remainder of the pathway known from pure cultures (e.g., *nahD*, *nahE*, salicylate hydroxylase large and small subunits, protocatechuate dioxygenase alpha, and beta chains) (Fig. 4; Data set S4 and S5). In addition, the MAG contained near-complete chemotaxis and flagella assembly, near-complete vitamin B_6_ and biotin biosynthesis, and a complete riboflavin biosynthesis pathway (Fig. S2). This organism has transporters for nitrite/nitrate, urea, phosphate, molybdate, and heme. Finally, it can transport nitrite and potentially reduce it to ammonium via a dissimilatory pathway (*nirBD*; Data set S4).

10.1128/mSystems.00245-21.2 FIG S2 Basic metabolic pathways in MAGs represented as fractions of the enzymes present. *Colwellia* and *Cycloclasticus* MAGs are labeled with a star. Download FIG S2, PDF file, 0.5 MB. Copyright © 2021 Sieradzki et al. 2021 Sieradzki et al. https://creativecommons.org/licenses/by/4.0/ This content is distributed under the terms of the Creative Commons Attribution 4.0 International license.

10.1128/mSystems.00245-21.4 FIG S4 Phylogenomic tree showing the placement of our *Colwellia* and *Cycloclasticus* MAGs. The MAGs are labeled in purple. The tree is based on 117 single-copy genes of *Gammaproteobacteria* and was built in GToTree. Complete reference genomes were downloaded from RefSeq on 20 August 2019. Download FIG S4, PDF file, 0.2 MB. Copyright © 2021 Sieradzki et al. 2021 Sieradzki et al. https://creativecommons.org/licenses/by/4.0/ This content is distributed under the terms of the Creative Commons Attribution 4.0 International license.

10.1128/mSystems.00245-21.8 DATA SET S3 Results of METABOLIC analysis of the 29 MAGs whose abundance increased in the presence of naphthalene. Each column represents a MAG, and each row represent a KEGG module. Download Data Set S3, XLSX file, 0.1 MB. Copyright © 2021 Sieradzki et al. 2021 Sieradzki et al. https://creativecommons.org/licenses/by/4.0/ This content is distributed under the terms of the Creative Commons Attribution 4.0 International license.

10.1128/mSystems.00245-21.9 DATA SET S4 Results of DRAM hits to open reading frames of the *Colwellia* MAG and *Cycloclasticus* MAG. Download Data Set S4, XLSX file, 0.5 MB. Copyright © 2021 Sieradzki et al. 2021 Sieradzki et al. https://creativecommons.org/licenses/by/4.0/ This content is distributed under the terms of the Creative Commons Attribution 4.0 International license.

The *Cycloclasticus* MAG (99% complete; 2.9% redundant, 2.4 Mbp) was detected in POLA naphthalene-amended metagenomes from May 2015, July 2014, and October 2014 (Fig. 3 B). This MAG was most closely related to Cycloclasticus zancles 78-ME (Fig. S4; Data set S3). It has a 78% average nucleotide identity (two-way ANI) and 81% average amino acid identity (two-way AAI) with the *Cycloclasticus* MAG assembled from the Deepwater Horizon oil spill (21). While it does not have the first steps of naphthalene degradation, it contains downstream genes of this pathway coding for 2-hydroxychromene-2-carboxylate isomerase (*nahD*), *trans*-*o*-hydroxybenzylidenepyruvate hydratase-aldolase (*nahE*), and catechol 2,3-dioxygenase (*xylE*) (Fig. 4; Data set S6). This MAG contains a near-complete flagellar assembly pathway but not the chemotaxis pathway. It can incorporate nitrogen from cyanate and biosynthesize riboflavin and biotin. This organism can degrade the aromatic hydrocarbon cymene and specifically contains the ring-opening enzyme *cmtC*. Similar to the *Colwellia* MAG, this MAG also contains transporters for nitrite/nitrate, urea, and phosphate and can potentially reduce nitrite to ammonium via *nirBD* (Data set S4).

Both MAGs contained secondary metabolite clusters of homoserine lactone (hserlactone) and lasso protein, which imply quorum sensing and a potential antimicrobial activity, respectively (35, 36). The *Cycloclasticus* MAG also contains clusters of bacteriocin and nonribosomal peptide synthase (NRPS), which may also point to a potential antibacterial activity.

### Metabolic characterization of MAGs enriched in the presence of naphthalene.

In addition to *Colwellia* and *Cycloclasticus*, 27 MAGs were significantly more abundant in naphthalene-amended samples (Wilcoxon rank test, *P* value < 0.05). One of these MAGs, Bin_47_1 (*Porticoccus*) contained a 16S-rRNA gene that matched at 100% identity to an OTU that was significantly enriched with naphthalene-derived ^13^C.

None of these MAGs contained the PAH or BTEX (benzene, toluene, ethylbenzene, xylene) degradation pathways defined by pure culture studies, but 12 of them had the KEGG pathway for degradation of the aromatic hydrocarbon cymene (Data set S4). However, 18 MAGs included an aromatic ring-hydroxylating dioxygenase. Nine of these MAGs can assimilate nitrogen from nitroalkanes (fuel additives) or nitriles. Finally, 24 out of these 29 MAGs contained at least 1 C1-oxidation enzyme, and 8 of the MAGs contained genes for oligosaccharide degradation, such as alpha-l-rhamnosidase, beta-xylosidase, and pullulanase (Data set S4).

Similar to *Colwellia* and *Cycloclasticus*, nine of these MAGs also contained secondary metabolite clusters of homoserine lactone (*Litoricola* Bin_27_2, *Rhodobacteraceae* POLA0714-12_bin_12), terpene (*Litoricola*, *Pseudohongiella*, *Rhodobacteraceae*, *Puniceispirillum*, SAR92, *Porticoccus*), and bacteriocin (*Rhodobacteraceae*).

Since several studies suggested that full degradation of PAHs may require bacterial consortia (21, 37), we also investigated the metabolic potential of the whole assembled community (open reading frames [ORFs] identified in contigs larger than 1 kbp). The full canonical naphthalene degradation pathway identified in pure cultures and incorporated into the KEGG database was not found even when combining all ORFs on contigs larger than 1,000 bp, whether they were binned into a MAG or not. In the pathway for degradation of naphthalene to benzoate or gentisate, we identified enzymes *nahA*, *nahD*, *nahE*, and salicylate hydroxylase, but not *nahB*, *nahC*, or salicylaldehyde dehydrogenase.

Moreover, to confirm that we did not miss additional naphthalene degradation genes because they did not assemble into contigs, we searched all forward reads from the POLA May 2015 ^13^C metagenome against all nucleotide sequences of naphthalene dehydrogenase ferredoxin subunit (*nahAc*, the first step of naphthalene degradation) from NCBI GenBank (188 references, September 2019). We chose this gene because it was not represented in the MAGs but is necessary for the function of naphthalene-1,2-dioxygenase. We found 248 reads (0.0008% of the total metagenome) that hit with a relaxed cutoff of E value 10^−3^ (mean identity, 97.8%; minimum bitscore, 30). Using a conservative estimate that this gene makes up 0.1% of the metagenome (similar to 16S-rRNA), and assuming one copy per cell and that each read maps to one copy of the gene, this would imply that only 8 out of 10,000 cells carry the naphthalene dioxygenase *nahAc* gene. This fraction would decrease even further if we dropped the single-copy and one read per gene assumptions.

In order to identify potential targets for biostimulation experiments, i.e., potentially limiting substances that might aid oil degradation if added after a spill, we performed a cross-comparison of the 29 MAGs that were significantly more abundant in naphthalene-amended water and searched for amino acid biosynthetic pathways, vitamin synthesis, potential nitrogen sources, and transporters.

While most MAGs had the ability to synthesize hydrophobic amino acids (leucine, isoleucine, valine, and proline) and some polar amino acids (cysteine, histidine, serine, and threonine), as well as charged amino acid lysine and amphipathic amino acid tryptophan, only one had the biosynthetic pathway for methionine, and none had biosynthetic pathways for arginine, phenylalanine, or tyrosine. However, 15 MAGs had a putative transport system for polar amino acids or branched amino acids, and 9 had a transport system for l-amino acids.

Only five MAGs contained a urea transport system, and while 34% of the naphthalene-enriched genomes had a urease gene, so did 43% of the genomes that did not respond to naphthalene amendment. In addition to *Colwellia* and *Cycloclasticus*, eight MAGs can degrade urea to ammonia via urease, and four MAGs have genes coding for *nirBD* nitrite reductase. Five MAGs contain a phosphonate transport system whereas twenty, including *Colwellia* and *Cycloclasticus*, can transfer phosphate.

Regarding vitamins, in addition to *Colwellia* and *Cycloclasticus*, 4 MAGs can synthesize biotin and 12 MAGs can synthesize pantothenate (vitamin B5). *Colwellia* and four additional MAGs can synthesize riboflavin (vitamin B2) (Fig. S2; Data set S4).

While oxidation of hydrocarbons is one source of energy for heterotrophic bacteria, the presence of light harvesting mechanisms may enhance their ability to grow and potentially incorporate naphthalene in ambient light conditions. Of the 29 MAGs with a significant response to naphthalene amendment, 5 are capable of anoxygenic photosynthesis (three *Rhodobacteraceae* and two *Halioglobus*), and 18 encode proteorhodopsins (Data set S4).

## DISCUSSION

### Naphthalene biodegradation detected only in the Port of Los Angeles.

Naphthalene degradation rates were detectable only at POLA but not at SPOT or CAT. As there is tidal mixing across the San Pedro Channel, we would expect some potential for degradation at those sites by bacteria advected from the port. A possible explanation for the nondetectable rates at SPOT and CAT is that diminishing PAH or other organic terrestrial runoff inputs offshore (38, 39) cannot support a degrader seed community compared to POLA. Indeed, the abundance of MAGs of the primary degraders *Colwellia* and *Cycloclasticus* was extremely low at CAT and SPOT. However, seasonality may also play a role in the detection of naphthalene degradation at SPOT and CAT. Some hydrocarbon-degrading bacteria, such as *Porticoccus*, live in association with algae that both synthesize and adsorb hydrocarbons (2). Had we collected samples in March or April, when algal blooms regularly occur in the San Pedro Channel, we might have been able to measure degradation outside POLA. These alga-associated hydrocarbon-degrading bacteria, even in pristine seawater, should be able to potentially respond to high input of hydrocarbons (2, 40, 41). Future studies of degradation rates could focus on sampling in the spring in order to confirm or refute this hypothesis in the San Pedro Channel.

The PAH carbon incorporation rate at POLA, however, indicated a minimum removal of ∼10% of the initial concentration per day. This rate is roughly 3-fold higher than that measured in seawater from the Gulf of Mexico amended with crude oil (7, 42), although the incubation time was shorter than for the Gulf of Mexico experiments, and the degradation rate may not be linear. The rate we measured is likely underestimated, as measurement was performed using GF/F filters, which have a pore size leading to the loss of roughly half of marine free-living prokaryotes. In addition, there could be degradation of PAH without incorporation of the labeled carbon into cells, which would not be accounted for by this measuring technique.

### Bacteria that responded to naphthalene amendment varied between sites and incubation conditions.

Incubation of POLA water with naphthalene revealed a difference in taxa that responded to naphthalene amendment after 24 h versus 88 h. First, it is noteworthy that many of the MAGs that were abundant in naphthalene-amended water after 88 h were already abundant after 24 h. Some of them were common marine heterotrophs such as *Puniceispirillum* (SAR116) and *Rhodobacteraceae*. Both *Pseudohongiella* and OM182 MAGs were matched by 16S-rRNA genes to OTUs that did not incorporate carbon from naphthalene, indicating that their increased abundance in the presence of naphthalene is not due to them being primary degraders. We speculate that their increased abundance in amended water is due to tolerance to naphthalene rather than utilization of naphthalene as a carbon source. We also note a difference in response time between the primary degraders. In three out of four 24-h incubations, *Cycloclasticus* abundance was already comparable to its abundance after 88 h, whereas *Colwellia* was only abundant in one 24-h incubation, and its abundance was orders of magnitude lower compared to 88 h. This may indicate a difference in growth rates and/or utilization of substrates and nutrients, as both taxa were undetectable in unamended water. Gutierez et al. (7) identified a 4- to 5-order of magnitude increase in the abundance of *Colwellia* and *Cycloclasticus* in PAH-amended seawater after 3 days. It is notable that the same naphthalene-degrading taxa appeared reproducibly in multiple incubations in different seasons. Thus, characterization of the metabolic requirements of these specific MAGs could be helpful in the future in case of an oil spill at the Port of LA.

Incubation conditions were not identical between the 24-h experiments from all three sites in July and October 2014 and the 88-h incubation (POLA only, May 2015). Incubation in the dark (88 h) would promote heterotrophy, while incubation in the light should still allow cyanobacteria and photosynthetic picoeukaryotes to compete over nutrients with potential naphthalene degraders. Sixteen of the MAGs that were enriched in amended 24-h incubations contained genes coding for proteorhodopsin. Proteorhodopsin can enhance ATP synthesis (43–45) and substrate uptake (45–47). Therefore, these organisms may have benefitted from incubation in light as opposed to dark conditions.

Some MAGs were abundant in amended water from SPOT and/or CAT even though there was no measurable degradation of naphthalene. They could be benefitting from the toxicity of naphthalene to other bacteria leading to release of dissolved organic carbon (48) or have the ability to degrade organic aromatic material which allowed them to utilize naphthalene in addition to other carbon sources. In the case of naphthalene utilization, we would have expected OTUs associated with these MAGs to be ^13^C-labeled. Only one of these MAGs, representing *Porticoccus*, a bacterium previously associated with PAH degradation (2, 49, 50), contained a 16S-rRNA gene. The matching OTU, which was 97% identical to the type strain Porticoccus hydrocarbonoclasticus (49), indeed showed minor ^13^C enrichment. However, it is plausible that these are naphthalene degraders that incorporated very little naphthalene. Low incorporation could happen either if naphthalene was not a main source of carbon and growth rates were fast enough to dilute the DNA labeling or, conversely, if these organisms are slow growers.

The duration of the 88-h SIP experiment may have enhanced accumulation of labeled carbon in bacterial DNA. In order to observe pronounced enrichment, the PAH degraders have to replicate at least once, and DNA density increases with every replication, as the new strand is made with labeled nucleotides. Additionally, it is methodologically difficult to observe enrichment in rare taxa (51). Since the primary degraders here were initially rare organisms, their abundance had to increase substantially before their enrichment could be tracked. In this study, there were 24 OTUs which did not belong to the main naphthalene degraders (*Colwellia*, *Cycloclasticus*, and *Neptuniibacter*) but were still significantly enriched (9 to 15%). While two of them (i.e., *Marinobacter* and *Pseudoalteromonas*) may be involved directly in degradation of naphthalene but either grow more slowly or were simply outcompeted by *Colwellia* and *Cycloclasticus* (52, 53), the rest are likely generalist heterotrophs which are also associated with degradation of algal blooms and the succession following them in the San Pedro Channel (e.g., *Flavobacteria*, Marine group A, SAR86) (54).

Cross-feeding is a common potential complication in the interpretation of SIP experiments in which by-products or end products of the labeled substrate are incorporated by organisms without the ability to degrade the original substrate. However, in the case of naphthalene degradation, incomplete degradation by primary degraders is less likely, as *Cycloclasticus* has been shown to completely degrade PAHs in culture (27), and both *Colwellia* and *Cycloclasticus* were heavily and comparably labeled. Generalists may have enzymes capable of degrading aromatic organic material, such as lignin, which have some affinity to naphthalene as well, leading to less enrichment compared to primary degraders due not to cross-feeding but to incorporation of carbon from various unlabeled substrates in addition to naphthalene.

### Metabolism of putative PAH-degraders and potential degraders.

In order to gain more insight into the metabolic requirements of naphthalene-degrading bacteria at POLA, we examined metabolic pathways and key transporters and enzymes within the annotated proteins in our MAGs. To date, only one published study characterized metabolic pathways in assembled genomes of marine oil-degrading bacteria, using metagenomes from naphthalene- and phenanthrene-amended seawater from the Deepwater Horizon (DWH) oil spill (7, 21). Within the DWH mesocosms, the prominent naphthalene degraders belonged to the genera *Cycloclasticus* and *Alteromonas*, and phenanthrene degraders included *Neptunomonas*, *Cycloclasticus*, and *Colwellia*, whereas we found *Cycloclasticus* and *Colwellia* to be the primary naphthalene degraders at POLA.

Both *Colwellia* and *Cycloclasticus* were nondetectable in both 16S-rRNA amplicons and MAG coverage before enrichment. However, after 88 h of incubation, they were the most dominant taxa in the mesocosms and exhibited very significant enrichment, indicating incorporation of naphthalene-derived carbon into their DNA over a substantial number of replication cycles.

The *Colwellia* MAG was the only one assembled here that contained two of the four annotated subunits of naphthalene 1,2-dioxygenase (*nahAa*, *nahAb*). As this is the first step of the pathway, requiring investment of reducing power (55), it is not surprising that several downstream enzymes as well as meta-cleavage of catechol are also present in the MAG. In comparison with the naphthalene degradation enzymes found in the *Colwellia* genome from the Gulf of Mexico, this MAG had different subunits of naphthalene dioxygenase as well as downstream enzyme *nahD* but lacked *nahB* and *nahF*, which were identified in the DWH MAG.

*Cycloclasticus* genes dominated the metagenome of the phenanthrene-enriched DWH mesocosm (21), highlighting its importance as a primary PAH degrader (56). Unlike the *Colwellia* MAG, our *Cycloclasticus* MAG contained hardly any part of the KEGG-defined aerobic naphthalene or phenanthreme degradation pathways which are based on pure cultures. This is in stark contrast to the *Cycloclasticus* genome assembled from DWH naphthalene-amended water (21). However, it did include several ring-hydroxylating dioxygenases. While these enzymes are best known to degrade single aromatic hydrocarbons, previous studies demonstrated that some single-ring aromatic degrading enzymes are capable of degrading naphthalene (two aromatic rings) efficiently (56–59). Additionally, *Cycloclasticus* has been shown to be able to degrade a variety of aromatics (27). Moreover, the *Cycloclasticus* MAG has a sigma-54-dependent transcription regulator with a potential hydrocarbon-binding domain as identified in *Cycloclasticus zancles* 78-ME (GenBank accession number AGS40441.1), which could control transcription of hydrocarbon degradation genes. Dioxygenases require iron and an iron-binding domain, such as ferredoxin, that can be shared by multiple enzymes (60, 61), which we found in this MAG. As we know that our *Cycloclasticus* phylotype can take up naphthalene-derived carbon as its main carbon source based on the SIP results and has genes that can participate in similar pathways but none of the culture-defined pathway, we posit that nontraditional dioxygenases with affinity to naphthalene were utilized to degrade naphthalene and downstream by-products. To further support this idea, metatranscriptomes of the microbial community within the oil plume of the Deepwater Horizon spill revealed low to nonexistent transcription of known PAH-degradation genes despite their presence in metagenomes (62). Moreover, the use of stable isotope probing indicated that both *Colwellia* and *Cycloclasticus* had the ability to degrade PAHs, whereas if only metagenomics was used, we might have concluded that they did not. The presence of C1-oxidizing enzymes in most naphthalene-enriched MAGS also implies that even those that cannot directly degrade naphthalene may benefit from degradation of by-products.

The presence of secondary metabolite clusters including lasso protein and bacteriocin in several MAGs suggests that part of the success of these organisms may be attributed to antimicrobial activity in addition to the ability to incorporate carbon from naphthalene.

### MAG-generated hypotheses for future biostimulation experiments at POLA.

Previous studies revealed that in many systems bioremediation of crude oil can be enhanced by addition of nitrogen and phosphorus (17, 63). While oil-degrading bacteria are found in many marine systems (64), their metabolic requirements may vary by system, depending on limitations *in situ*. The significant genomic dissimilarity between the strains of *Colwellia* and *Cycloclasticus* found at POLA and the ones found at the Gulf of Mexico implies that we cannot assume similarity in metabolic requirements between systems. As oil degradation requires a considerable amount of nitrogen (65), choosing the correct form of nitrogen could be crucial. The same could apply to other nutrients.

Both *Colwellia* and *Cycloclasticus* displayed a potential for using a variety of nitrogen sources to different ends, with a full dissimilatory nitrate reduction to ammonium (DNRA) pathway and nitrite/nitrate transporters (*focA*, *narK*), as well as a urea transporter and the ammonium-assimilating glutamate synthase pathway. Nitrate, nitrite, and ammonium are always detectable at POLA in surface seawater (66) and thus are not limiting nutrients in this site. It is possible that PAH degradation occurs in large part on particles that contain anaerobic microniches at POLA, which would explain the presence of DNRA within the MAGs. *Colwellia* and *Cycloclasticus* strains have also been isolated from sediments, where conditions may become anaerobic and support DNRA (9, 12). However, ammonium assimilation via glutamate synthase is an aerobic process that could be sustained in seawater.

Based on the functional pathways and ABC transporters found in MAGs of naphthalene incorporators, we propose specific targets for future experiments on enhancement of PAH bioremediation at the Port of Los Angeles. Phosphate is more likely to augment bioremediation than phosphonate. To supply iron for dioxygenase synthesis, adding heme/hemoproteins should be superior to adding Fe(II) or Fe(III), as 27/29 enriched MAGs contain heme transporters. Marine bacterioplankton have been shown to be able to incorporate iron from heme groups (67, 68). Finally, polar amino acids, for which we did not find any biosynthetic pathways but did find transporters in enriched MAGs, could also potentially augment bioremediation.

In the current climate of excessive use of fossil fuels, chronic deposition of toxic and recalcitrant polycyclic aromatic hydrocarbons into the coastal ocean is inevitable. PAH-degrading bacteria may provide some control over the remineralization of these inputs and could serve as targets for bioremediation technologies. Identification of naturally occurring biodegraders is a crucial first step, but optimization of the degradation process requires knowledge of the metabolic requirements of local organisms (26). Moreover, their genomic information remains an available resource should other hypotheses for biostimulation arise.

## MATERIALS AND METHODS

### Field sites and sample collection.

While the Port of Los Angeles (POLA) is not routinely monitored for PAHs, it is located in an area with natural oil seeps, it houses a ship-refueling station of high-aromatic-content marine diesel (Environmental Science and Technology Centre [https://etc-cte.ec.gc.ca/databases/OilProperties/pdf/WEB_Marine_Diesel_Fuel_Oil.pdf], 22 November 2017), and it is surrounded by the second-largest metropolitan area in the United States, which is likely a source of consistent atmospheric PAH deposition. The LA-Long Beach port is the busiest port in the United States and the 10th-busiest in the world according to the International Association of Ports and Harbors (IAPH [https://www.iaphworldports.org], 26 November 2017). A report published in 2010 revealed detectable levels of many PAHs at various stations within the port and as far as outside the port entrance, corresponding to our sampling site (69, 70). Due to the high marine traffic at POLA, there is constant resuspension into the water column of sediment, which would normally (without such regular resuspension) serve as a sink for PAHs due to their tendency to attach to particles (69, 70). The port of LA consistently has higher nutrient concentrations compared to SPOT and CAT, with the ammonium concentration ranging seasonally between 0.1 and 3 μM and NOx between 0.2 and 2 μM (66).

Surface seawater was collected in July and October 2014 from three sites across the San Pedro Channel near Los Angeles, CA, USA (Fig. 1)—the port of Two Harbors, Santa Catalina Island (CAT), the San Pedro Ocean Time-series (SPOT), and the Port of Los Angeles (POLA). An additional sample was collected in May 2015 only from POLA. Water was collected by high-density polyethylene (HDPE) bucket into two 10-liter low-density polyethylene (LDPE) containers and stored in a cooler in the dark until arrival at the lab.

### Isotope addition and incubation.

Unlabeled (^12^C) naphthalene or fully labeled ^13^C-10-naphthalene (ISOTEC, Miamisburg, OH, USA) as crystals at a maximum final concentration of 400 nM was added to each 10-liter container of water collected without replication. This concentration is roughly 3 orders of magnitude lower than the solubility of naphthalene in water (30 mg/liter = 234 μM). Ammonium-chloride at a final concentration of 1 μM was also added to each container to prevent nitrogen limitation of PAH degraders. Naphthalene-amended seawater from all three sites from July and October 2014 was incubated in 10% ambient light, which is comparable to the light at 5-m depth at SPOT, at surface water temperature (17°C) for 24 h. POLA water from May 2015 was incubated in the dark at surface water temperature (20°C) for 88 h. Incubation of POLA water in the dark was performed to simulate more closely the conditions in that site, as light attenuation at POLA is much steeper than that of the other sites (Fig. S1). At the end of the incubation period the seawater was filtered through an 80-μm mesh and a glass fiber Acrodisc (Millipore-Sigma, St. Louis, MO, USA) prefilter (pore size 1 μm) followed by a 0.2-μm polyethersulfone (PES) Sterivex filter (Millipore-Sigma) to capture only planktonic bacteria and archaea. After filtration, 1.5 ml sodium-chloride-Tris-EDTA (STE; 10 mM Tris-HCl, 1 mM EDTA and 100 mM NaCl) buffer was injected into the Sterivex casing, and the filters were promptly sealed and stored in –80°C.

10.1128/mSystems.00245-21.1 FIG S1 Relative photosynthetically active radiation (PAR) profiles at the Port of Los Angeles (POLA), the San Pedro Ocean Time-series (SPOT), and Santa Catalina Island (CAT) in spring (April), winter (January), and fall (October). Download FIG S1, PDF file, 0.2 MB. Copyright © 2021 Sieradzki et al. 2021 Sieradzki et al. https://creativecommons.org/licenses/by/4.0/ This content is distributed under the terms of the Creative Commons Attribution 4.0 International license.

### Carbon incorporation rate measurement.

Seawater (2 liter per bottle, with headspace) from all three sites was incubated in quadruplicate 2-liter polycarbonate bottles with 400 nM ^13^C labeled naphthalene. Ammonium-chloride at a final concentration of 1 μM was also added to each bottle, similar to the previously described incubations. A single bottle from each site was filtered immediately after amendment to establish a t_0_ atom percent ^13^C of the particulate carbon as a baseline for incorporation rate measurement. The remaining bottles (3 replicates per site) were incubated in a temperature-controlled room (see above). Incubations were carried out for ∼24 h and were terminated by filtration of microbial biomass onto precombusted (∼5 h at 400°C) 47-mm GF/F filters (Whatman, Maidstone, United Kingdom). The filters were then dried at 60°C and kept in the dark until analysis. Isotopic enrichment was measured on an IsoPrime continuous-flow isotope ratio mass spectrometer (CF-IRMS). IRMS data were corrected for both size effect and drift before being calculated as previously described (71).

### DNA extraction.

Total DNA was extracted from the Sterivex filters using a modified DNeasy plant kit protocol (Qiagen, Hilden, Germany). The Sterivex filters containing STE buffer were thawed; 100 μl 0.1 mm glass beads was added into the filter casing and put through two 10-min cycles of bead beating in a TissueLyser device (Qiagen, Hilden, Germany). The flowthrough, pushed out using a syringe, was incubated for 30 min at 37°C with 2 mg/ml lysozyme followed by another 30-min incubation at 55°C with 1 mg/ml proteinase K and 1% SDS. The resulting lysate was loaded onto the DNeasy columns, followed by the protocol described in the kit instructions. Only samples from the POLA May 2015 sample yielded enough DNA for ultracentrifugation of both the labeled and unlabeled DNA. However, metagenomes were sequenced from all dates and sites except SPOT October 2014, as the amount of extracted DNA was insufficient for library preparation.

### Ultracentrifugation and density-fraction retrieval.

Isopycnic ultracentrifugation and gradient fractionation were performed as described in previous work (25, 72). Briefly, DNA from the labeled and unlabeled samples was added into separate quick-seal 5-ml tubes (Beckman Coulter, Indianapolis, IN, USA) combined with 1.88 g/ml CsCl and gradient buffer for a final buoyant density of 1.725 g/ml. The tubes were sealed and centrifuged in a Beckman Optima L100 XP ultracentrifuge and near-vertical rotor NVT 65.2 at 44,100 rpm (190,950 relative centrifugal force [rcf]) at 20°C for 64 h.

The gradient was divided into 50 fractions of 100 μl each. Refraction was measured using 10 μl of each fraction using a Reichert AR200 digital refractometer and converted into buoyant density (ρ = 10.927 · n_c_-13.593) (73). DNA in each fraction was preserved in 200-μl polyethylene glycol (PEG) and 1 μl glycogen, precipitated with ethanol, eluted in 50 μl Tris-EDTA (TE) buffer, and quantified using PicoGreen (Invitrogen, Carlsbad, CA, USA).

### Amplification of the 16S-rRNA V4-V5 hypervariable regions.

PCR was performed on each fraction with detectable DNA. Each reaction tube contained 12 μl 5 Prime HotMaster mix (QuantaBio, Beverly, MA, USA), 1 μl (10 pg) barcoded 515F-Y forward primer (5′-GTG**Y**CAGCMGCCGCGGTAA), 1 μl (10 pg) indexed 926R reverse primer (5′‐CCGYCAATTYMTTTRAGTTT), 1 ng DNA, and 10 μl molecular-grade water.

Thermocycling conditions were as follows: initial denaturation at 95°C for 3 min, 30 cycles of denaturation at 95°C for 45 s, annealing at 50°C for 45 s, and elongation at 68°C for 90 s, followed by a final elongation step at 68°C for 5 min.

PCR products from each fraction were cleaned using 1× Agencourt AMPure XP beads (Beckman Coulter), quantified with PicoGreen, and diluted to 1 ng/μl. A pool of 1 ng of each uniquely barcoded product was cleaned and concentrated again with 0.8× Agencourt AMPure XP beads.

The pooled amplicons were sequenced on an Illumina MiSeq instrument (University of California at Davis, USA) for 600 cycles. Each pool was also spiked with 1 ng of an even and a staggered mock community in order to assess the sequencing run quality (74). All expected OTUs were found in the observed mock communities and accounted in total for 99.5% of the reads, indicating that there was no unexpected bias in the sequencing run (75). Sequencing yield information can be found in Data set S1.

10.1128/mSystems.00245-21.6 DATA SET S1 *P* values of Kolmogorov-Smirnov normality test of ^12^C- and ^13^C-naphthalene-enriched OTU distribution as a function of density, density shift, corresponding atom percent excess, and SILVA-assigned taxonomy of 180 OTUs analyzed from POLA 5/15. Download Data Set S1, XLSX file, 0.02 MB. Copyright © 2021 Sieradzki et al. 2021 Sieradzki et al. https://creativecommons.org/licenses/by/4.0/ This content is distributed under the terms of the Creative Commons Attribution 4.0 International license.

### Amplicon data processing.

The raw reads were quality-trimmed using Trimmomatic (76) version 0.33 with parameters set to LEADING:20 TRAILING:20 SLIDINGWINDOW:15:25 MINLEN:200 and merged with Usearch version 7 (77) with a limit of a maximum of 3 differences in the overlapping region. The resulting merged-reads were analyzed in mothur (78) and clustered at 99% identity following the MiSeq standard operating procedures (SOP) (20 March 2016) (79) with one exception; we found that degapping the aligned sequences, aligning them again, and dereplicating them again fixed an artifact in which a few abundant OTUs were split due to the alignment, despite 100% identity, due to an additional terminal base between the OTU representative sequences. OTUs with a total of less than 10 reads over all fractions were removed from the analysis. The remaining 2,366 OTUs were assigned taxonomy using the arb-silva SINA search and classify tool version 1.3.2 (80).

### Detection of OTU enrichment due to substrate incorporation.

Plots of normalized abundance as a function of density were generated in R (https://www.r-project.org/) for the top 200 most abundant OTUs in each sample. The abundance of each OTU was normalized to a sum of 1 across all fractions.

To detect enrichment, the weighted mean density of an OTU in the labeled and unlabeled samples was calculated, and if the difference exceeded 0.005 g/ml, the shift was determined to be significant (22). As quantitative SIP (qSIP) is sensitive to OTU abundance (22, 51), OTUs were used further only if they were nonspurious OTUs, whose distribution could not be differentiated from a normal distribution in both enriched and control samples (Kolmogorov-Smirnov test, alpha = 0.05). With these criteria, 180 OTUs were further analyzed.

### Metagenomic library preparation.

The original unfractionated DNA extracted from the Sterivex filters was sheared with a Covaris m2 instrument to a mean length of 800 bp. Libraries were prepared from 15 ng of sheared DNA using the Ovation ultralow DR multiplex system v2 kit (NuGEN, Redwood City, CA, USA) with 9 amplification cycles. The libraries were bead purified as described above and sequenced on an Illumina MiSeq device for 600 cycles (UC Davis, USA) or on an Illumina HiSeq rapid run for 500 cycles (University of Southern California [USC] genome core). See Data set S1 for a detailed list of sequenced metagenomes.

### Metagenomic sequencing analysis.

Reads were quality-trimmed using Trimmomatic version 0.33 as described above. Paired reads were assembled per sample in an iterative subsampling and assembly process as described in Hug et al. (81) but using metaSPAdes version 3.9.1 instead of IDBA-UD, followed by overlap assembly with minimus 2 with minimum overlap of 200 bp and minimum identity of 99%. Paired reads from all sequenced samples (Table S1) were mapped back to the contigs with BBmap (sourceforge.net/projects/bbmap/) requiring 95% identity. Binning was performed using two approaches: (i) binning the POLA 5/15 13C metagenome with CONCOCT (82) and bin refinement in Anvi’o (83) and (ii) pooling contigs longer than 5 kbp from four naphthalene-enriched metagenomes (POLA 5/15 12C, POLA 5/15 13C, POLA 7/14 12C, POLA 7/14 13C), dereplicating them with cd-hit at 99% id (84, 85), and binning them using a combination of MaxBin 2 (86), CONCOCT (82), and MetaBAT 2 (87). These bins were combined, refined, and reassembled using the MetaWRAP pipeline (88). The resulting metagenome-assembled genomes (MAGs) generated by both methods were dereplicated using dRep (89), and only MAGs that were at least 50% complete and under 10% redundant were analyzed.

10.1128/mSystems.00245-21.5 TABLE S1 Sample details—number of metagenomics (MG) reads and 16S-rRNA amplicons. *, Numbers of amplicons in enriched mesocosms are combined across all density fractions. Download Table S1, DOCX file, 0.01 MB. Copyright © 2021 Sieradzki et al. 2021 Sieradzki et al. https://creativecommons.org/licenses/by/4.0/ This content is distributed under the terms of the Creative Commons Attribution 4.0 International license.

Initial taxonomic assignment of MAGs was performed using GTDB-Tk (90). We then improved the taxonomy by generating class-level phylogenomic trees with GToTree (33) using NCBI RefSeq complete genomes and placing the bins assigned to the class by GTDB-Tk within them.

Reads from additional unenriched seawater metagenomes from all three sites were also mapped to the dereplicated MAG set to detect MAGs whose abundance increased in the presence of naphthalene. 16S-rRNA genes found in MAGs were searched against the amplicon OTUs.

### Metabolic analysis.

Open reading frames (ORFs) in the final set of metagenome-assembled genomes (MAGs) were predicted using Prodigal (91) and annotated by assignment to clusters of orthologous groups (COGs) using the Anvi’o anvi-run-ncbi-cogs function. KEGG (Kyoto Encyclopedia of Genes and Genomes) orthology for ORFs was assigned with KofamScan using the prokaryote profile and its built-in thresholds (92). KofamScan results were summarized using KEGGDecoder (93) (Fig. S2). The taxonomic classification of ORFs was determined based on lowest-common ancestry using Kaiju (94) and based on the RefSeq database.

Secondary metabolite clusters were identified using the antiSMASH bacterial version 5 online platform (95) with the “relaxed cutoffs” option. Iron-related transport and storage systems were identified using FeGenie (96). Additional functional analysis was also done with METABOLIC version 1.3 (97) and DRAM (98).

Read recruitment from different samples to the MAGs and viral contigs was analyzed with Anvi’o (83) using the Q2Q3 setting. This setting ignores the 25% lowest covered and 25% highest covered positions within the MAG when calculating mean coverage to avoid bias due to islands or highly conserved genes.

### Data availability.

Metagenomic and amplicon raw reads from enrichment mesocosms can be found on EMBL-ENA under project PRJEB26952 and samples ERS2512855 to ERS2512864. The metagenomic library blank is under sample ERS2507713. Amplicon reads can be found under sample accession numbers ERS2507470 to ERS2507679, and PCR blanks and mock communities, under samples ERS2507702 to ERS2507712. Metagenomic t_0_ raw reads can be found under project PRJEB12234 and samples ERS2512914 to ERS2512919.
