# Supplementary figures and images for: Metagenomics and Quantitative Stable Isotope Probing Offer Insights into Metabolism of Polycyclic Aromatic Hydrocarbon Degraders in Chronically Polluted Seawater
Source: mSystems. 2021 May 11;6(3):e00245-21. doi: 10.1128/mSystems.00245-21 (PMC8125074; doi:10.1128/mSystems.00245-21)

Mean coverage Q2Q3

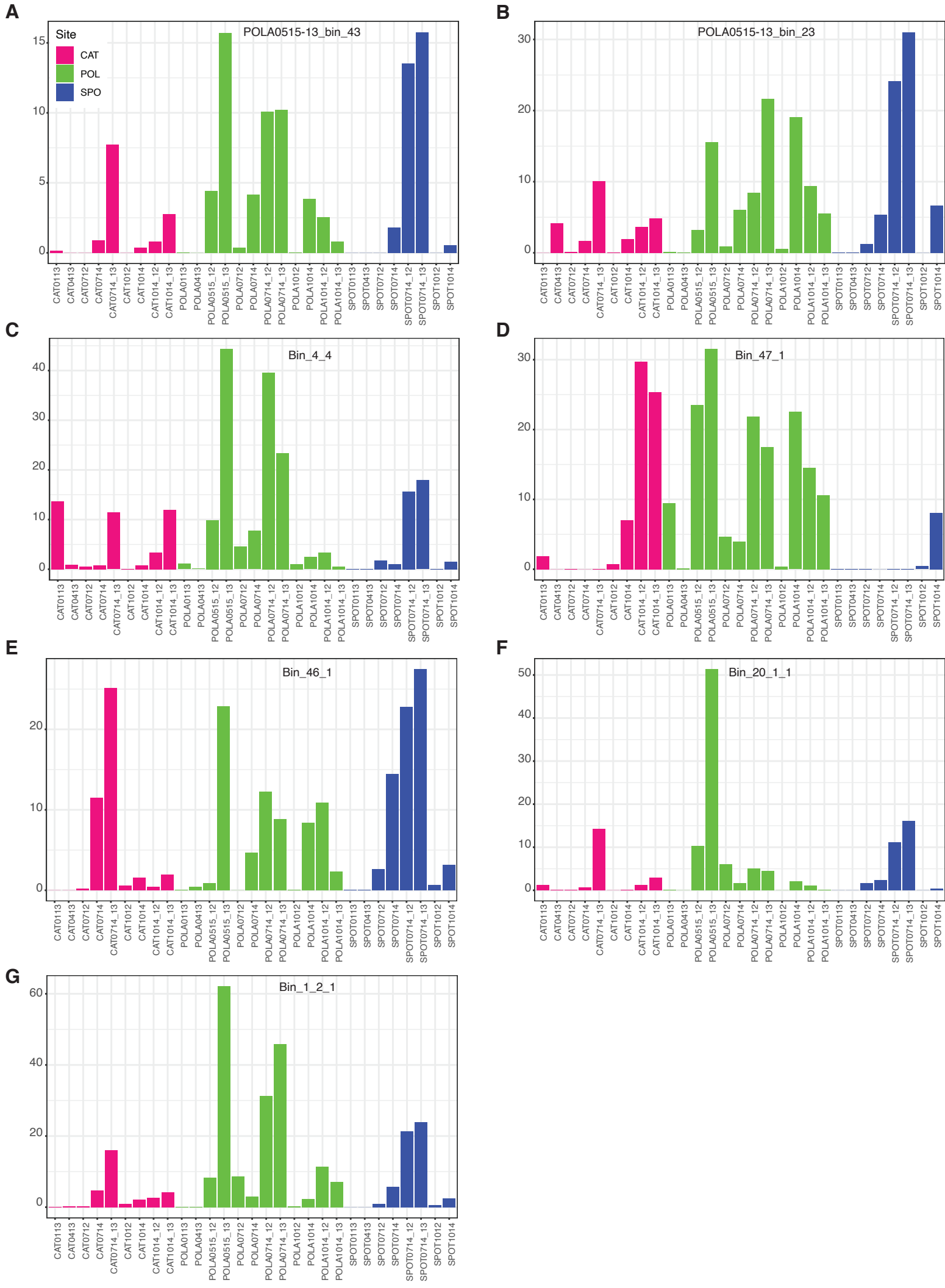

Supplement: FIG S3 [file mSystems.00245-21-sf003.pdf]

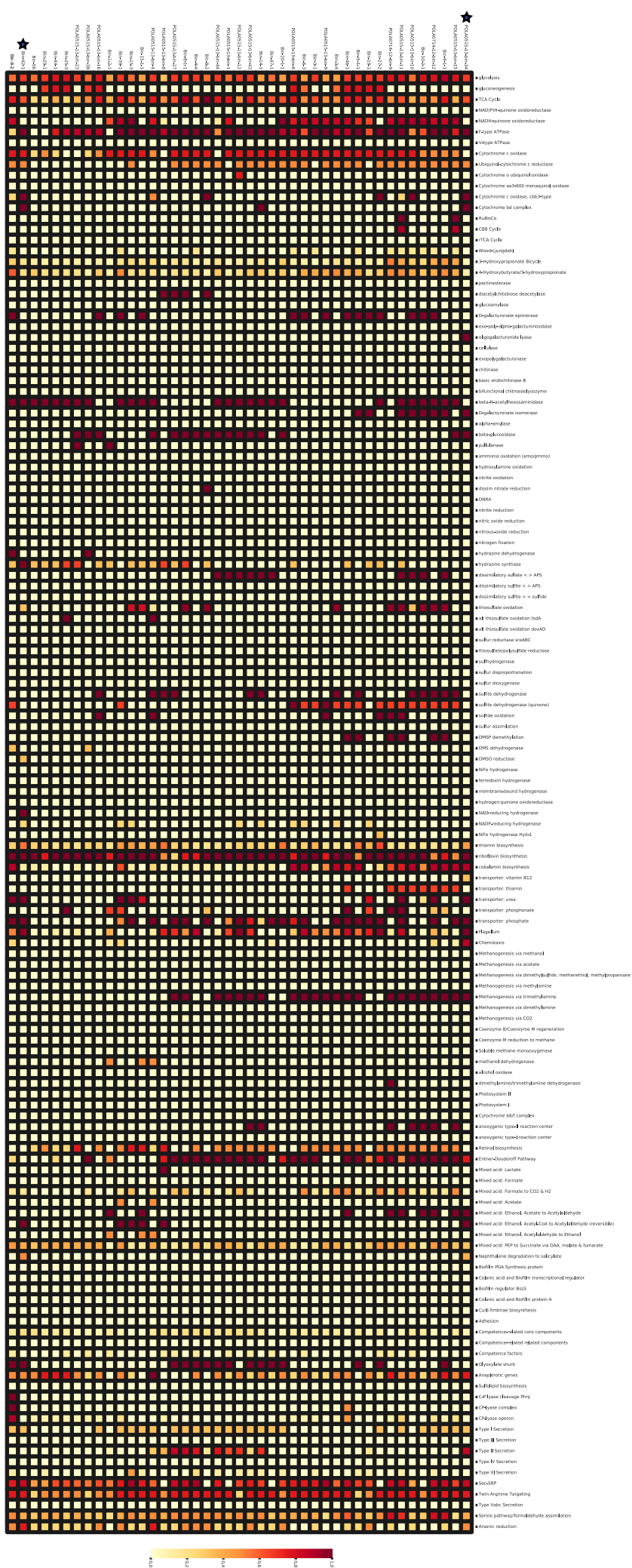

Supplement: FIG S2 [file mSystems.00245-21-sf002.pdf]

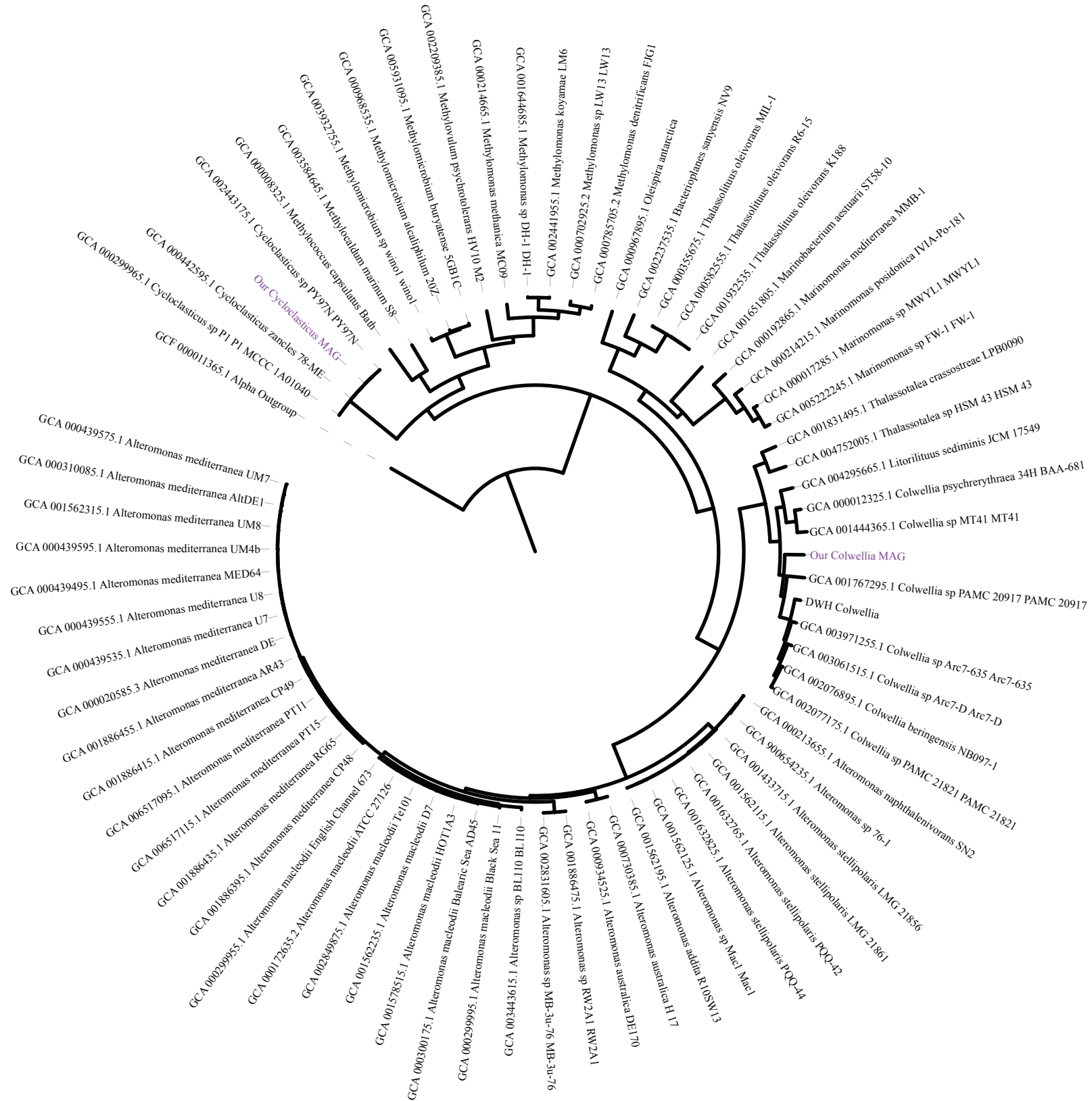

Supplement: FIG S4 [file mSystems.00245-21-sf004.pdf]

10/19/2012 DOBD Relative PAR Profiles

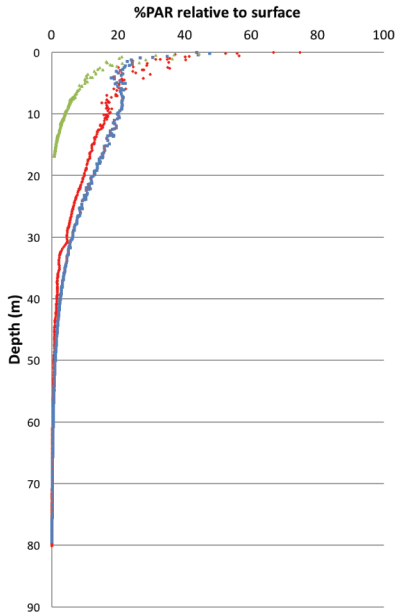

04/24/2013 DOBD Relative PAR profiles

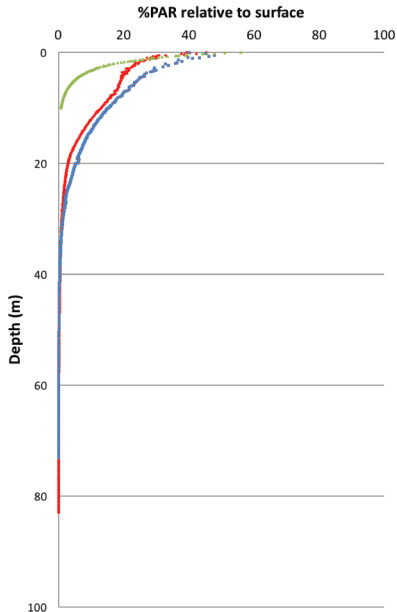

01/09/2013 DOBD Relative PAR Profiles

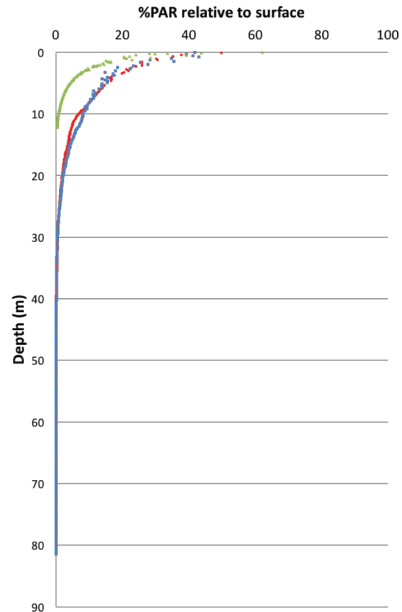

Supplement: FIG S1 [file mSystems.00245-21-sf001.pdf]
